# Long noncoding RNA LINC00511 induced by SP1 accelerates the glioma progression through targeting miR‐124‐3p/CCND2 axis

**DOI:** 10.1111/jcmm.14331

**Published:** 2019-04-11

**Authors:** Chen Li, Hongjiang Liu, Jipeng Yang, Jiankai Yang, Liang Yang, Yuanyu Wang, Zhongjie Yan, Yuchen Sun, Xiaofeng Sun, Baohua Jiao

**Affiliations:** ^1^ Department of Neurosurgery The Second Hospital of Hebei Medical University Shijiazhuang P.R. China

**Keywords:** CCND2, glioma, LINC00511, miR‐124‐3p, SP1

## Abstract

Mounting evidence suggests the vital roles of long noncoding RNA (lncRNAs) in the glioma. However, the role of LINC00511 in gliomagenesis is still uncovered. Here, in this study, we aim to investigate the effects of LINC00511 on the glioma cancer phenotype and its deepgoing mechanism. Results indicated that LINC00511 was up‐regulated in glioma tissues and cell lines, moreover its overexpression positively correlated with the poor prognosis and advanced pathological stages. For the upstream regulation, LINC00511 was epigenetically up‐regulated by transcription factor specificity protein 1 (SP1). Gain and loss of functional experiments demonstrated that LINC00511 promoted the proliferation and invasion of glioma cells in vitro. The knockdown of LINC00511 repressed the tumour growth in vivo. Mechanistically, LINC00511 positively regulated the CCND2 expression via competitively sponging with miR‐124‐3p. Overall, our finding illuminates that LINC00511 is induced by SP1 and accelerates the glioma progression through targeting miR‐124‐3p/CCND2 axis, constructing the SP1/LINC00511/miR‐124‐3p/CCND2 axis.

## 1 INTRODUCTION

Glioma is the most common malignant intracranial tumour, accounting for 50%‐60% of brain malignancies, and causes the potential crisis for the human health worldwide.^1^, ^2^ Regarding the histological category of glioma, there are astrocytoma, glioblastoma, oligodendroglioma, mixed oligoastrocytomas and pilocytic astrocytoma.^3^, ^4^ Regarding the histopathological features classification released by World Health Organization (WHO), glioma is also classified to be grades I and II, which are low, and grades III and IV, which are advanced.^5^ Although the greatest efforts have been invested for the glioma therapy, including the surgical resection followed by chemoradiotherapy, the post‐operative prognosis is still negative and whether certain therapeutic methods could inhibit glioma progression need to be addressed clearly.^6^

Emerging studies have demonstrated the increasing vital roles of noncoding RNAs (ncRNAs) in the human diseases, especially cancers.^7^, ^8^ For the glioma, conservatively, hundreds of lncRNAs have been reported to participate in the cellular processes modulation. For instance, lncRNA CASC9 is highly expressed in glioma specimens and the ectopic overexpression modulates the proliferative ability, invasion in vitro and mechanically sponged miR‐519d/STAT3 positive feedback loop.^9^ Furthermore, DLX6‐AS1 accelerates the tumour phenotype of glioma via miR‐197‐5p targeting the E2F1 gene, constructing DLX6‐AS1‐miR‐197‐5p‐E2F1 axis.^10^ These evidence shows the diverse cellular pathophysiological process in the glioma, such as differentiation, metastasis and angiogenesis.

Long intergenic noncoding RNA 00511 (LINC00511) has been regarded as oncogene in several human cancers, such as tongue squamous cell carcinoma, pancreatic ductal adenocarcinoma and non‐small‐cell lung cancer.^11^, ^12^ Herein, we discover the oncogenic role of lncRNA LINC00511 in the glioma cellular progression, whose overexpression is motivated by the transcription factor specificity protein 1 (SP1). LINC00511 enhance the tumourous phenotype via sponging the miR‐124‐3p and targeting CCND2 production, thereby providing a model for LINC00511‐mediated cellular regulation in glioma.

## 2 MATERIALS AND METHODS

### 2.1 Patients and specimens

Fresh tissue samples from the surgical extraction, including glioma and adjacent non‐cancerous tissue specimens, were stored in liquid nitrogen and collected at The Second Hospital of Hebei Medical University. All the tissue samples were diagnosed with glioma by pathologists. Patients enrolled in this research were aware of the study and signed the informed consent.

### 2.2 Cell and small interfering RNA transfection

The human glioma cell lines (U87, U251, SHG44, A172) were purchased from the American Type Culture Collection (ATCC, Manassas, VA) as well as normal human astrocytes (NHA). Cell were cultured with RPMI‐1640 medium (Gibco, Carlsbad, CA), supplemented with 10% foetal bovine serum (FBS, Gibco, Carlsbad, CA) and 100 U/mL penicillin/streptomycin (Life Technologies, CA) in humidified incubator with 5% CO_2_ at 37°C. Small interfering RNA (siRNA) was synthesized by GenePharma Company (Shanghai, China). The transfection of siRNA (si‐LINC00511 and negative controls) was separately transfected into glioma cells using Lipofectamine 2000 reagent (Invitrogen, Carlsbad, CA). All the sequences were showed in the Table S1.

### 2.3 Real‐time quantitative PCR

Total RNA was isolated from glioma cells or tissues using TRIzol (Invitrogen, Carlsbad, CA) based on the guiding of manufacturer's specification. RNA quantity was determined by a NanoDrop2000 spectrophotometer (Thermo Scientific, Waltham, MA). For qRT‐PCR, RNA (1 μg) was reverse transcribed to cDNA using a reverse transcription kit (Takara, Dalian, China). SYBR Green PCR Master Mix (Applied Biosystems, Carlsbad, CA) and miRNA qRT‐PCR detection kit were used for the PCR on an ABI7500 Real‐time PCR instrument (Applied Biosystems). GAPDH was measured as an internal control. The primers for LINC00511, miR‐124‐3p and CCND2 were listed in Table S1. Relative expression was calculated using the 2^‐ΔΔCT^ method.

### 2.4 CCK‐8 and colony formation assay

The proliferation of glioma cells was tested using CCK‐8 assay and colony formation assay according to manufacturer's instructions. CCK‐8 agent (Dojindo, Rockville) was administrated into the 96‐well plates at a density of 2 × 10^3^ per well. The absorbance was recorded at 450 nm using microplate reader. Glioma cells were cultured in RPMI1640 containing 10% FBS and seeded into the 6‐well plates at 2 × 10^3^ per well for 2 weeks. Then, the cells were washed and fixed with methanol and stained with 0.5% crystal violet. The colonies greater than 150 μm were counted under a microscope.

### 2.5 Transwell invasion assay

The transwell invasion assay was conducted using the matrigel‐coated chambers (BD Biosciences, San José, CA) with 8‐µm pores. Cells (2 × 10^5^) were seeded in the upper chambers with in serum‐free medium. The medium in the lower chamber was added 10% FBS. After 48 hours of incubation at 37°C, the non‐invaded cells were scraped with a cotton swab. The cells were fixed with 10% formalin and stained with 0.1% crystal violet for 30 minutes.

### 2.6 Western blot analysis

Western blotting was performed as described. Cell lysates were extracted using RIPA protein extraction reagent buffer (Sigma‐Aldrich, St Louis, MO) containing a protease inhibitor cocktail and phenylmethylsulfonyl fluoride (Roche, CA). Anti‐SP1 and anti‐cyclin D2 polyclonal antibodies (1:1000, Abcam, Cambridge, MA) were used for blotting. GAPDH (1:1000, Abcam) was used as a loading control.

### 2.7 Cellular cytoplasm/nucleus fraction isolation

The nuclear and cytosolic fractions were conducted using a PARIS Kit (Life Technologies, Carlsbad, CA, USA) as previously described.^14^

### 2.8 Luciferase reporter assay

The LINC00511 and CCND2 3′‐UTR sequences containing the wild‐type or mutant miR‐124‐3p binding sites were synthesized. These sequences were cloned into pmirGLO luciferase reporter vector (Promega) and transfected into the U251 cells with miR‐124‐3p mimics or control using Lipofectamine 2000. The luciferase activities were measured after 48 hours by the dual‐luciferase reporter assay kit (Promega).

### 2.9 Chromatin immunoprecipitation

Glioma cells (U251) were used for the Chromatin immunoprecipitation (ChIP) assay according to the instructions of the EZ‐ChIP™ Chromatin immunoprecipitation kit (Millipore, USA). Cells were treated with formaldehyde to generate DNA‐protein cross‐links. Then, cells were sonicated to produce 200 to 300 bp chromatin fragments. Anti‐SP1 specific antibody (Millipore) was applied for immunoprecipitations and IgG was used as the negative control. The precipitated chromatin DNA was analysed by qRT‐PCR.

### 2.10 Tumour formation in vivo

The tumour formation in vivo assay was carried out in BALB/c nude mice (male, 4 to 6 weeks old). After the transfection of shRNA targeting LINC00511 and empty vector, 1 × 10^6^ U251 cells were subcutaneously injected into the flanks to establish xenograft model. Animal experiment was carried out in accordance with the Guide for the Care and Use of Laboratory Animals. The tumour size was monitored using calipers by measuring the length and width. The volumes were calculated using the formula: (length × width^2^)/2.

### 2.11 Statistical analysis

The differences within each group were determined using Student's *t* test or one‐way ANOVA. The data or value were analysed by mean × standard deviation (SD). Patient survival rate was calculated by the Kaplan‐Meier plot and Cox proportional hazards model. A two‐sided *P* < 0.05 was considered statistically significant.

## 3 RESULTS

### 3.1 LINC00511 overexpression indicated the poor clinical outcome of glioma

**Figure 1:**
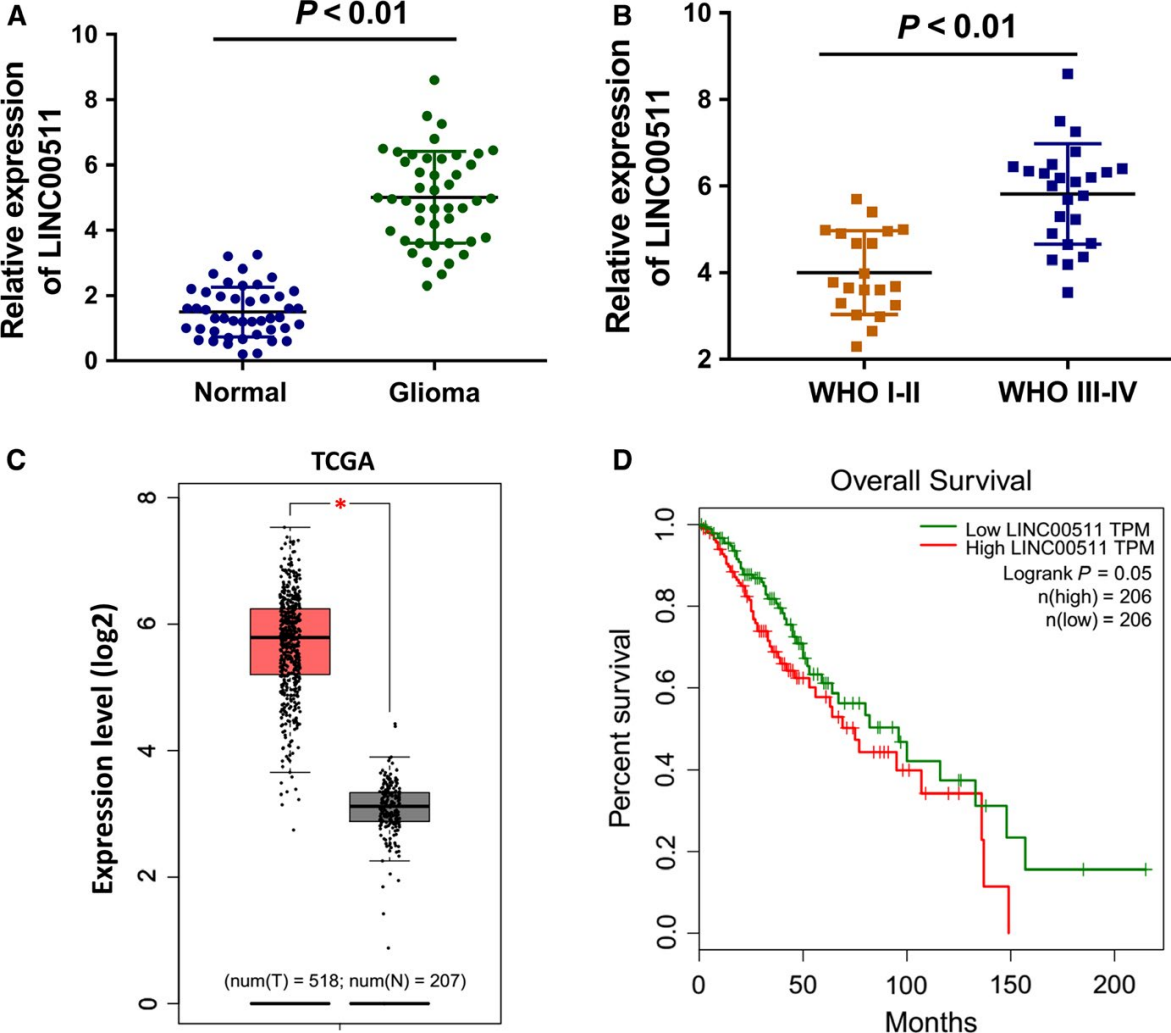
LINC00511 overexpression indicated the poor clinical outcome of glioma. (A) RT‐PCR demonstrates the expression level in the glioma tissue specimens and adjacent normal tissue. (B) LINC00511 expression in the advanced pathological type (WHO III‐IV) and the primary pathological type (WHO I‐II). (C) The data set based on the TCGA data (http://gepia.cancer-pku.cn/) demonstrated the LINC00511 level in the glioma samples (n = 518) and the control (n = 207). (D) Overall survival analysis based on the TCGA showed the survival rate of glioma patients with higher or lower level of LINC00511

Clinically, the expression of LINC00511 was measured using the RT‐PCR in tissue samples, demonstrating the high expression level in the glioma tissue specimens (Figure 1A). What's more, the expression of LINC00511 was much more up‐regulated in the advanced pathological type (WHO III‐IV) than the primary pathological type (WHO I‐II) (Figure 1B). In larger sample size analysis, the Gepia data set based on the TCGA data (http://gepia.cancer-pku.cn/) demonstrated that LINC00511 was more higher in the glioma samples (n = 518) than the control (n = 207) (Figure 1C). The overall survival analysis based on the TCGA showed that the patients with higher level of LINC00511 indicated the lower survival rate compared to the lower group (Figure 1D). Those data suggest that LINC00511 overexpression indicated the poor clinical outcome of glioma.

### 3.2 Transcription factor SP1 accelerates the transcription of LINC00511 in glioma cells

**Figure 2:**
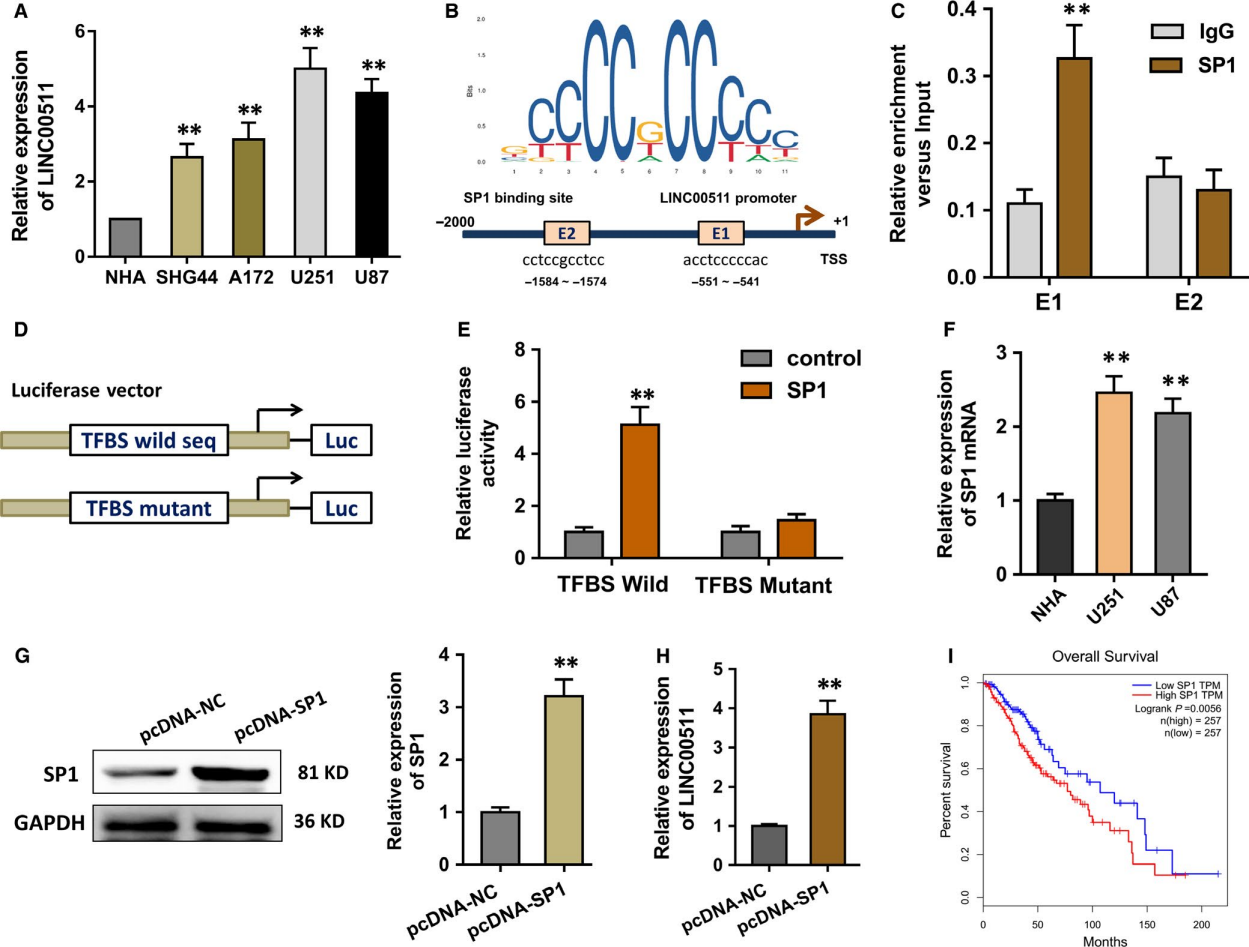
Transcription factor SP1 accelerates the transcription of LINC00511 in glioma cells. (A) The expression level of LINC00511 in the glioma cells detected by the RT‐PCR. (B) Schematic diagram for the potential binding sites for transcription factor SP1 with the promoter of LINC00511 using the online bioinformatics tools (JASPAR, http://jaspar.genereg.net/). (C) Chromatin immunoprecipitation (ChIP) assay revealed the transcriptional activation of two element (−1584 ~ −1574, −551 ~ −541). (D) The luciferase reporter vector, including wild‐type and mutant type, was constructed using the U251 cells. (E) Luciferase reporter assay showed the activities of the cotransfection of vector and SP1. (F) SP1 mRNA was tested by the PCR. (G) The SP1 protein level in the U251 cells transfected with enhanced SP1 overexpression plasmid. (H) LINC00511 RNA level in the U251 cells transfected with enhanced SP1 overexpression plasmid. (I) The survival analysis of SP1 indicated the poor prognosis of glioma patients based on the TCGA database. **P < 0.01

The expression level of LINC00511 was not only highly expressed in the glioma tissues, but also overexpressed in the glioma cells detected by the RT‐PCR (Figure 2A). In the investigation of the upstream regulation of LINC00511, we found that transcription factor SP1 could bind with the promoter of LINC00511 using the online bioinformatics tools (JASPAR, http://jaspar.genereg.net/) (Figure 2B). The more detailed context was supplemented in supplementary materials. There were two potential binding sites on the promoter region (E1, E2). ChIP assay revealed that SP1 could directly bind to the first element (E1, −551 ~ −541) which was responsive to the SP1‐mediated transcriptional activation (Figure 2C). Then, the luciferase reporter vector for the region, including wild‐type and mutant type, was constructed and transfected into the U251 cells (Figure 2D). Results demonstrated that the transfection of TFBS (transcription factor binding sites) wild‐type had the marked activities compared to the mutant types (Figure 2E). SP1 mRNA tested by the PCR revealed its up‐regulation in the glioma cells (U87, U251) (Figure 2F). The enhanced SP1 overexpression plasmid could increase not only the SP1 protein level (Figure 2G), but also the LINC00511 RNA level (Figure 2H). The survival analysis based on the TCGA database demonstrated that the high level of SP1 indicated the poor prognosis of glioma patients (Figure 2I). Therefore, data confirm that the transcription factor SP1 accelerates the transcription of LINC00511 in glioma cells.

### 3.3 LINC00511 promoted the proliferation, invasion and tumour growth of glioma cells

**Figure 3:**
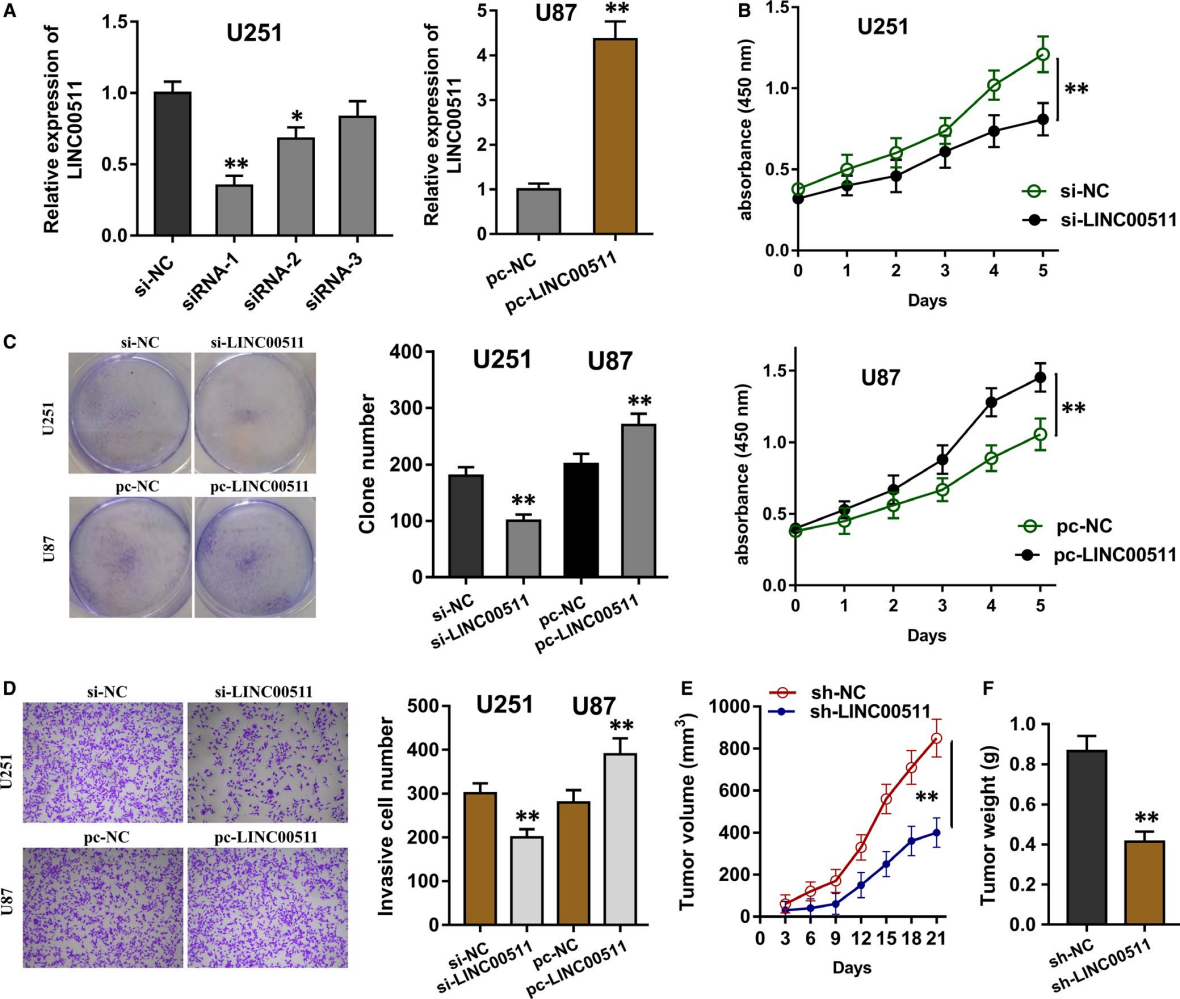
LINC00511 promoted the proliferation, invasion and tumour growth of glioma cells. (A) The silencing (siRNA) and overexpression plasmid (pcDNA3.1‐LINC00511) of LINC00511 were constructed. (B) CCK‐8 assay determined the proliferative ability of glioma cells (U251, U87) transfected with the siRNA and plasmid for LINC00511. (C) Clone formation assay showed the acceleration of LINC00511 overexpression plasmid for the clone formation and the inhibition of LINC00511 silencing. (D) Transwell assay for the invasion indicated the invaded cells of LINC00511 overexpression plasmid and INC00511 silencing. (E, F) The tumour growth of glioma cells in vivo transfected with LINC00511 silencing using U251 cells. **P < 0.01

In the following cellular assay, the silencing and overexpression of LINC00511 were constructed to test its roles on the tumourous phenotype of glioma cells (Figure 3A). CCK‐8 assay was performed to determine the proliferative ability of glioma cells (U251, U87), demonstrating the activated proliferation of glioma cells by the LINC00511 (Figure 3B). Clone formation assay was performed to detect the colony formation, showing the acceleration of LINC00511 overexpression plasmid transfection for the clone formation and the inhibition of LINC00511 silencing (Figure 3C). Transwell assay for the invasion was carried out and indicated the stimulative invaded cells of LINC00511 overexpression plasmid and the inhibition of LINC00511 silencing (Figure 3D). In vivo, the LINC00511 silencing could repress the tumour growth of glioma cells (Figure 3E,F). Therefore, LINC00511 promoted the proliferation, invasion and tumour growth of glioma cells.

### 3.4 LINC00511 epigenetically sponges the miR‐124‐3p in the glioma cells

**Figure 4:**
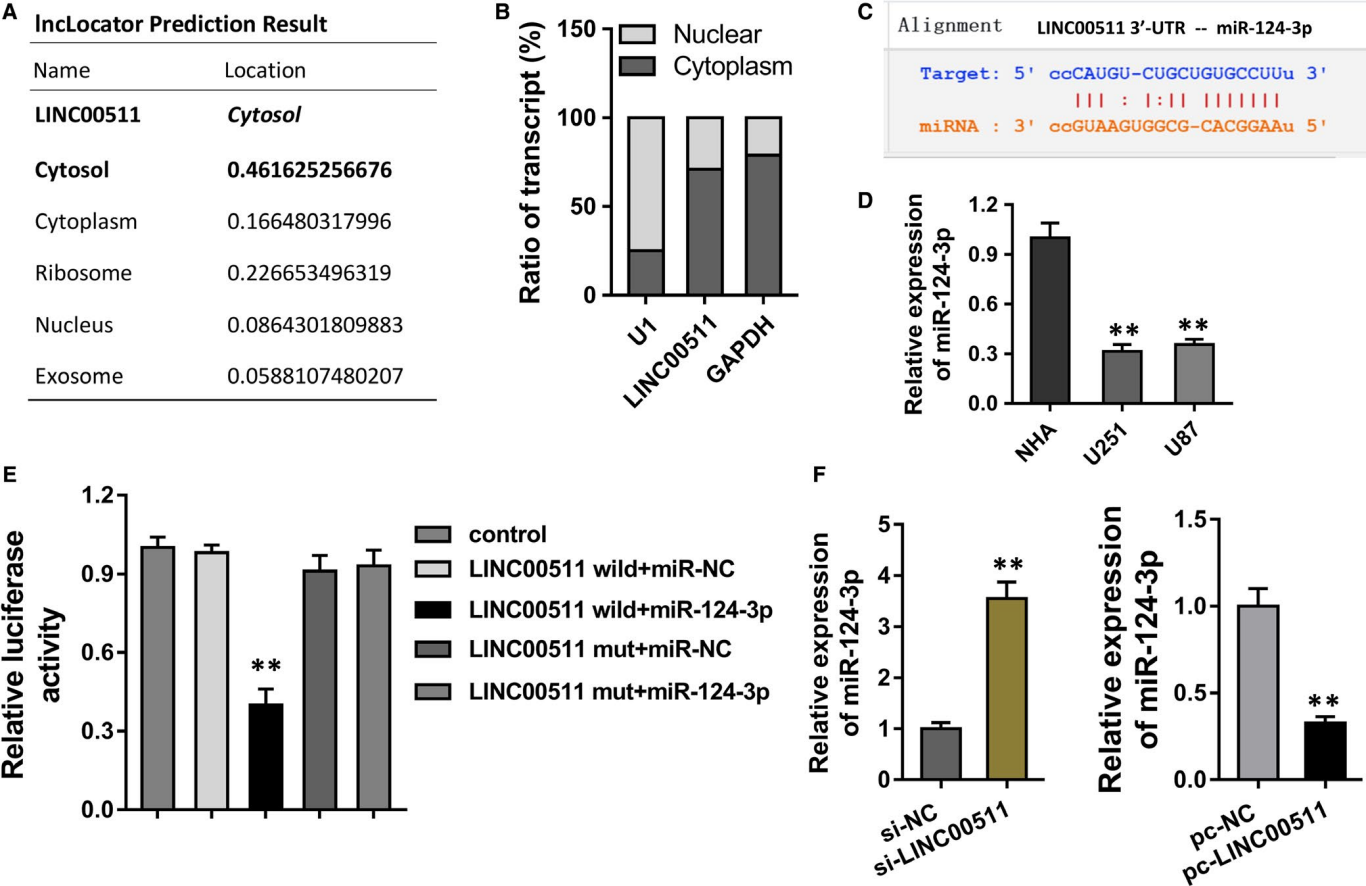
LINC00511 epigenetically sponges the miR‐124‐3p in the glioma cells. (A) Database lncLocator (http://www.csbio.sjtu.edu.cn/bioinf/lncLocator/) showed the location of lncRNA LINC00511 in the cytosol and cytoplasm of glioma cells (U251). (B) Subcellular fractionation indicated the subcellular distribution of LINC00511 in the glioma cells. (C) Database StarBase (http://starbase.sysu.edu.cn/) showed the molecular binding within the miR‐124‐3p and LINC00511 located in the 3′ Untranslated Regions (3′‐UTR). (D) RT‐PCR showed the miR‐124‐3p in the U251 and U87 cells. (E) Luciferase reporter assay indicated the luciferase activity after the transfected into U251 cells. (F) miR‐124‐3p level in the U251 cells after the transfection of LINC00511 siRNA and LINC00511 plasmid. **P < 0.01

The deepgoing mechanism by which LINC00511 regulates the glioma tumourous phenotype was investigated. Database lncLocator (http://www.csbio.sjtu.edu.cn/bioinf/lncLocator/) showed that lncRNA LINC00511 was mainly located in the cytosol and cytoplasm of glioma cells (Figure 4A). Subcellular fractionation indicated that the subcellular distribution of LINC00511 in the glioma cells was a cytoplasmic element (Figure 4B). Database StarBase (http://starbase.sysu.edu.cn/) showed the molecular binding within the miR‐124‐3p and LINC00511 located in the 3′ Untranslated Regions (3′‐UTR) (Figure 4C). RT‐PCR showed that the miR‐124‐3p was presented the low expression in the U251 and U87 cells (Figure 4D). Then, the evidence by luciferase reporter assay clearly indicated the reduced luciferase activity after the luciferase vector was transfected into U251 cells (Figure 4E). In the U251 cells, the transfection of LINC00511 siRNA up‐regulated the miR‐124‐3p, while the transfection of LINC00511 plasmid reduced the miR‐124‐3p (Figure 4F). So, the data suggest that LINC00511 epigenetically sponges the miR‐124‐3p in the glioma cells.

### 3.5 CCND2 is modulated by the LINC00511 and miR‐124‐3p

**Figure 5:**
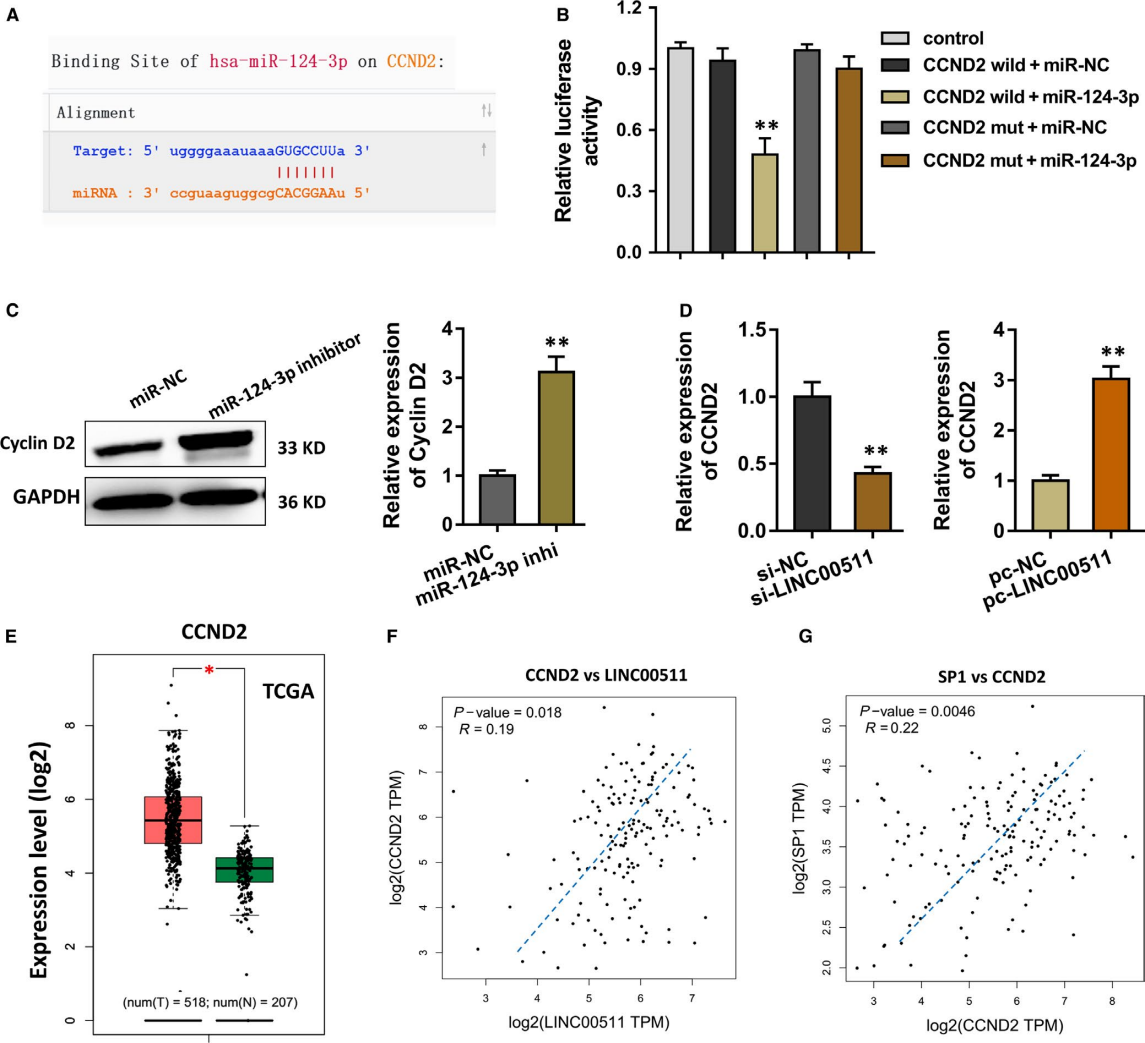
CCND2 is modulated by the LINC00511 and miR‐124‐3p. (A) The identification within LINC00511 and miR‐124‐3p, we subsequently discover the possible target for miR‐124‐3p in the glioma carcinogenesis. Starbase 3.0 (http://starbase.sysu.edu.cn/) remaindered the complementary sites in CCND2 gene with miR‐124‐3p. (B) The evidence by luciferase reporter assay indicated the reduced luciferase activity of CCND2 wild‐type and miR‐124‐3p transfected in U251 cells. (C) Western blot analysis revealed the cyclin D2 protein, encoded by the CCND2 gene, in the miR‐124‐3p inhibitor transfection. (D) The CCND2 mRNA level in the transfection of LINC00511 siRNA and the LINC00511 plasmid. (E) The data based on the TCGA demonstrated the overexpression of CCND2 level in the glioma tissue samples. (F, G) Spearman correlation coefficient analysis indicated the positive correlation within CCND2 and LINC00511, and the SP1 and CCND2. **P < 0.01. *P < 0.05

After the identification within LINC00511 and miR‐124‐3p, we subsequently discover the possible target for miR‐124‐3p in the glioma carcinogenesis. Starbase 3.0 (http://starbase.sysu.edu.cn/) remaindered us that the CCND2 gene could have the complementary sites with miR‐124‐3p (Figure 5A). Then, the evidence by luciferase reporter assay once again indicated the reduced luciferase activity after the luciferase vectors, CCND2 wild‐type and miR‐124‐3p, were transfected into U251 cells (Figure 5B). Western blot analysis revealed that the cyclin D2 protein, encoded by the CCND2 gene, was increased in the miR‐124‐3p inhibitor transfection (Figure 5C). Moreover, the transfection of LINC00511 siRNA could reduce the CCND2 mRNA level, however, the LINC00511 plasmid activated it (Figure 5D). The data based on the TCGA demonstrated that the CCND2 level was noticeably overexpressed in the glioma tissue samples (Figure 5E). And the correlations were measured by Spearman correlation coefficient analysis indicated the positive correlation within CCND2 and LINC00511 (Figure 5F), and the SP1 and CCND2 (Figure 5G). Therefore, CCND2 is modulated by LINC00511 and miR‐124‐3p.

## 4 DISCUSSION

The rapid development of high‐throughput genomics sequencing has discovered thousands of novel disease‐associated lncRNAs.^15^ It is worth noting that increasing evidence has indicated the wild functions by which lncRNAs accelerate or inhibit the glioma tumourigenesis.^16^, ^17^ On the other hand, lncRNAs also could function as a promising prognostic marker in glioma.^18^

The lncRNA LINC00511 has been identified to be oncogene in the human cancers, for example non‐small‐cell lung cancer, breast cancer and ductal adenocarcinoma.^19^ In the carcinogenesis of glioma, the detailed mechanism is still unclear. Here, this research illustrated that LINC00511 was markedly increased in the glioma tissue specimens and cells. More adequately, the ectopically high levels indicate the bad outcome of glioma patients with high level of LINC00511. The cellular assay in vitro and in vivo revealed that the LINC00511 could promote the proliferative ability and invaded ability of glioma cell, as well as the tumour growth. Therefore, the data could confirm the tumour facilitator of LINC00511 for gliomagenesis.

To investigate the upstream of LINC00511, we found that the transcription factor SP1 could bind with the promoter region of LINC00511, thereby activating the transcriptional level and enrich the abundance. The abundance of SP1 is verified to be increased in the glioma cells, and its overexpression is also correlated with the poor prognosis. In the pancreatic ductal adenocarcinoma, Zhao X et al (2018) reported that LINC00511 sponges hsa‐miR‐29b‐3p, acting as a competing endogenous RNA, to regulate VEGFA expression.^13^ This finding sparks the inspiration that LINC00511 might regulate the glioma genesis via the competing endogenous RNA regulation. The preliminary experimental results suggest that the subcellular location of LINC00511 is located in the cytoplasm and cytosol, therefore, indicating the potential post‐transcriptional regulation of LINC00511. miRNA‐124‐3p was found to be interacted with LINC00511 3′‐UTR, which was confirmed by the luciferase reporter assay.

More and more evidence have confirmed the anti‐cancer role of miRNA‐124‐3p in human cancers, such as the bladder cancer, pancreatic ductal adenocarcinoma, cervical cancer and so on.^20^, ^21^ In the glioma genesis, it is also reported that miRNA‐124‐3p exerts its tumour inhibitor role via targeting the target protein.^23^ Therefore, we confirm the oncogenic role of LINC00511, besides results also verify the anti‐cancer role of miRNA‐124‐3p in the glioma. Further results reveal that CCND2 is modulated by the LINC00511 and miR‐124‐3p. CCND2 and its encoded cyclin D2 protein both regulate the cellular progression of glioma cells.^24^

Transcriptional factor specificity protein 1 (SP1) has been found to be up‐regulated in the glioma and participate the glioma genesis. For example, SP1 binds to the MDK gene promoter and directly promotes MDK expression, showing the SP1‐MDK axis cooperated in glioma tumourigenesis.^25^ More and more lncRNAs have been identified to participate in the gliomagenesis with increasing evidence,^26^, ^27^ such as, lncRNA FOXD2‐AS1 is up‐regulated in glioma tissue/cells and CCND2 is also up‐regulated. Then, the up‐regulation of CCND2 was closely correlated with poor prognosis of glioma patients and CCND2 knockdown suppresses the cellular proliferation, migration, invasion and EMT in glioma cells via miR‐185‐5p and FOXD2‐AS1 regulation.^28^ Another example, NEAT1 and CDK6 could promote the proliferation and metastasis of glioma cells and inhibit cell apoptosis, while the miR‐139‐5p suppresses the tumour effect on the biological functions of glioma cells.^29^

In summary, our study discovered the LINC00511 overexpression, inspired by the transcription factor SP1, in the glioma cells. Moreover, the up‐regulation of LINC00511 competitively sponges the miR‐124‐3p, thereby motivating the CCND2 and its encoded cyclin D2 protein expression, which might likely provide great promise for glioma therapeutics.

## CONFLICT OF INTEREST

All authors declare no conflict of interest.

## AUTHORS' CONTRIBUTIONS

Chen Li, Hongjiang Liu, Jipeng Yang, Jiankai Yang performed the assays. Liang Yang, Yuanyu Wang, Zhongjie Yan, Yuchen Sun assist these assays. Chen Li, Baohua Jiawrite and revise the paper.
