# Supplementary material for: Long noncoding RNA LINC00511 induced by SP1 accelerates the glioma progression through targeting miR‐124‐3p/CCND2 axis
Source: J Cell Mol Med. 2019 Apr 11;23(6):4386–94. doi: 10.1111/jcmm.14331 (PMC6533561; doi:10.1111/jcmm.14331)
Supplement: Supplementary file 1 [file JCMM-23-4386-s001.docx]

**Supplement Table 1**. Primers sequences for qRT-PCR and sequences of siRNA.

|  | Sequences |
| --- | --- |
| LINC00511 | forward, 5’-CGCAAGGACCCTCTGTTAGG-3’,  reverse, 5’-GAAGGCGGATCGTCTCTCAG-3’ |
| miR-124-3p | forward, 5’-TGTGATGAAAGACGGCACAC-3’  reverse, 5’-CTTCCTTTGGGTATTGTTTGG-3’ |
| CCND2 | forward, 5’-ACCTTCCGCAGTGCTCCTA-3’  reverse, 5’-CCCAGCCAAGAAACGGTCC-3’ |
| GAPDH | forward, 5’-AGAAGGCTGGGGCTCATTTG-3’  reverse, 5’-AGGGGCCATCCACAGTCTTC-3’ |
